# Knowledge, Attitude, and Practice of Antibiotic Resistance Among General Population in Saudi Arabia: A Cross-Sectional Study

**DOI:** 10.7759/cureus.51053

**Published:** 2023-12-24

**Authors:** Lulwah S Alkhuraisi, Hyder Mirghani, Mohammed M Al Qahtani, Wafa A Alrezqi, Ibrahim A Alfaifi, Abdulrahman S Jathmi, Abdulaziz S Jathmi, Nawal S Aianazi

**Affiliations:** 1 College of Medicine, University of Tabuk, Tabuk, SAU; 2 Internal Medicine, University of Tabuk, Tabuk, SAU; 3 College of Medicine, Al-Baha University, Al-Baha, SAU; 4 College of Medicine, Jazan University, Jazan, SAU; 5 College of Medicine, Northern Border University, Arar, SAU

**Keywords:** saudi arabia, practice, attitude, knowledge, antibiotic resistance

## Abstract

Background: Antibiotic resistance is a global public health concern, and understanding the knowledge, attitudes, and practices (KAP) of the general population is essential for effective prevention and management. This cross-sectional study aimed to assess the KAP of antibiotic resistance among adults in Saudi Arabia.

Materials and methods:Between August 2023 and October 2023, 1000 participants aged 16-65 years from various regions in Saudi Arabia were surveyed using an online questionnaire. Data were analyzed using Excel and IBM SPSS Statistics version 27.0.0 (Armonk, NY: IBM Corp.). The sociodemographic characteristics of the participants were examined, and KAP variables towards antibiotic resistance were explored through a range of statistical methods, including frequencies, percentages, means, and standard deviations. Significance was defined as a p-value of ≤0.05.

Results:The majority of participants were Saudi natives (98.1%) with ages between 16 and 25 years (38%), and over half were female (55.7%). More than half held a university degree (54.7%), mainly in non-medical fields (73.3%), and a significant portion reported a monthly income above 10,000 Saudi Riyals (49.7%). Regarding knowledge, 76.5% were aware of antibiotic resistance, but only 24.2% correctly identified its causes. Attitude assessments showed that 50.8% never used antibiotics as a preventive measure, and 47.3% always followed medical prescriptions. Practices revealed that 50.5% rarely used antibiotics, and 68.8% obtained antibiotic prescriptions from doctors. Additionally, 68.9% stopped taking antibiotics only after completing the course.

Conclusion:It is important to implement health education campaigns aimed at the public, emphasize the role of health care providers in health education for the general public, and enforce stringent regulations to control the non-prescription dispensing of antibiotics. However, further studies are needed on this subject in the future.

## Introduction

Before the development of antibiotics, infectious diseases were the major causes of death globally with an average life expectancy at birth of 46 years for males and 48 years for females [1]. An antibiotic is a chemical substance secreted from one organism and is toxic to another organism [2]. A unique mode of action of an antibiotic affects bacterial survival, but more critically, at therapeutic concentrations [3]. Antibiotic resistance has emerged as a global public health concern, and it is currently spreading quickly. The risk of patient treatment failure, mortality, and healthcare expenses rises with multidrug resistance [4]. Unregulated drug supply, insufficient surveillance, and a pervasive attitude toward antimicrobial misuse, including self-medication, are the primary causes of the rise in antimicrobial resistance [5]. Antimicrobial resistance is a pressing public health issue since it is thought to contribute to at least 700,000 fatalities annually worldwide and more than 35,000 deaths annually in the United States. Around 3% of people in developed countries and about 100% of people in developing countries are believed to use antibiotics without a prescription [6]. An estimated 4.95 million fatalities in 2019 were attributed to antibiotic resistance. According to global research, if no further intervention is done, antibiotic resistance would kill 10 million people annually and around 100 trillion USD would be lost by 2050 [7].

The incidence of resistant bacteria among Gram-negative bacteria ranges from 7.6% to 92.3%, with *Pseudomonas aeruginosa*, Acinetobacter species, and *Klebsiella pneumoniae* having the highest prevalence. Furthermore, the incidence of resistance among Gram-positive bacteria ranges from 23.5% to 30.7%, with methicillin-resistant *Staphylococcus aureus* (MRSA), *Clostridium difficile,* and *Streptococcus pneumoniae* having the highest prevalence according to a review of studies conducted in Saudi Arabia between 1990 and 2011 [8]. Approximately one-third of all antibiotic prescriptions are unnecessary [9]. In 2019, a study was conducted in the Hail region, Saudi Arabia, among 500 participants and the results showed that the percentage of awareness about antibiotic resistance was 46.6%, 17.2% had doubts about antibiotic resistance, and 36.2% were unaware of the existence of antibiotic resistance [10]. In 2019, a study was conducted in a private university in Malaysia and included medical students from all years, the study aimed to evaluate the understanding and awareness of antibiotic resistance. The results have shown that except for 32.7% of students in the preclinical period, the majority of students were aware that antibiotics are only used to treat bacterial infections and not viral infections [11]. A study was conducted in Sweden with 1000 randomly collected samples and aimed to assess the level of knowledge about antibiotic usage. The results showed that 19.1% agreed that antibiotics are effective in treating a common cold, 77.2% of respondents concurred that antibiotics are better for bacterial infections and 80.7% of respondents agreed that microorganisms may develop drug resistance [12]. This study aimed to measure the level of knowledge, attitude, and practice toward antibiotic resistance in Saudi Arabia.

## Materials and methods

Study design, setting, and population

This is a cross-sectional study. The target population includes the general population in Saudi Arabia from different regions including the Northern, Southern, Eastern, and Western regions. The sample in our study included adults aged between 16 and 65 years from both genders living in Saudi Arabia.

Sampling technique and sample size

A simple random sampling technique was used to obtain participants from different regions in Saudi Arabia, the target population included adults aged between 16 and 65 years from both genders. Using the Raosoft calculator (Seattle, WA: Raosoft, Inc.), a sample size of 385 was estimated with a confidence level of 95%. The size of the sample was calculated by using the following formula: n=P (1-P) *Zα^2^/d^2^ with a 95% confidence level. Here, n is calculated sample size, Z is z-value for the selected level of confidence (1-a)=1.96, P is an estimated prevalence of knowledge, Q=(1-0.50)=50% (i.e., 0.50), and d is the maximum acceptable error (0.05). So, the calculated minimum sample size was n=(1.96)^2^ × 0.50 × 0.50/(0.05)^2^=384.

Recruitment procedure and inclusion and exclusion criteria

The data was collected via a self-administrated, electronic Google Forms questionnaire and was distributed by data collectors from different regions in Saudi Arabia through social media applications. Participants in our study had to electronically give their consent before participation, then we used the inclusion and exclusion criteria to obtain our target population. Adults (male and female) aged between 16 and 65 years, living in Saudi Arabia, and who agreed to participate were included in this study. Children younger than 16 years and/or adults older than 65 years and/or not living in Saudi Arabia were excluded from the study.

Consent and outcome measures

Consent was obtained electronically before starting to fill out the self-administered online questionnaire. Consent from either the adults or legal guardians was mandatory before participants below the age of 18 began filling out the questionnaire. The level of knowledge, attitude, and practice toward antibiotic resistance among the general population in Saudi Arabia were the outcome measures of this study.

Data collection procedure

Data for our study was collected using an electronic self-administered questionnaire which was designed by Google Forms, then it was distributed electronically via data collectors through social media applications. The questionnaire was created based on previous relevant studies conducted in Saudi Arabia, the authors of those studies were contacted and consulted (table in appendix) [13,14]. Individuals in our study had to electronically provide their consent before participation. Our questionnaire consists of four sections of closed-ended questions. The first section contains the sociodemographic data, the second one will evaluate the knowledge about antibiotic resistance, the third one will assess the attitude toward antibiotic use and the fourth one will assess the practice of antibiotic usage. The questionnaire is available in Arabic and English languages. The data has been collected in the period from August 2023 to October 2023.

Data monitoring and analysis

Investigators are responsible for keeping the security of the data and informing the participants that the data were not to be used for any other purpose except for this study. Also, personal data such as name and contact information were not included in the questionnaire. Our data were first indexed in Microsoft Excel which were extracted directly from Google Forms questionnaire results. The data were analyzed using IBM SPSS Statistics version 27.0.0 (Armonk, NY: IBM Corp).

Ethical considerations and potential risks

Approval for the research was obtained by the Local Research Ethics Committee (LREC) at Tabuk University, Tabuk, Saudi Arabia, with ethical approval number UT-305-147-2023. Informed consent was obtained from participants before started filling out the questionnaire. The participants were informed that participation was entirely voluntary and that no personal information would be requested. The study carries no expected risks to the participants.

## Results

A comprehensive statistical analysis was performed on the dataset, encompassing a wide array of both descriptive and inferential methods. The study explored the sociodemographic characteristics of the participants and their knowledge, attitude, and practice (KAP) variables towards antimicrobial resistance, by calculating simple frequencies and percentages, and the results were systematically presented in tables.

The responses to knowledge questions were systematically tabulated with numbers and percentages, with accurate answers indicating a good level of knowledge about antimicrobial resistance. To assess attitudes, Likert scales with the following four response options were employed: "never" (coded as 1), "sometimes" (coded as 2), "often" (coded as 3), and "always" (coded as 4). The collected data were analyzed by calculating the mean (±SD) response for each attitude question, utilizing the assigned numerical values. In terms of practices related to antibiotic resistance, the prevalence of responses among participants was presented in terms of numbers, percentages, and a pie chart. Statistical significance was defined as having a p-value of 0.05 or lower, and a 95% confidence interval was computed for the analyses. The data were analyzed using IBM SPSS Statistics version 27.0.0.

The study involved 1000 participants from the general population in Saudi Arabia, with the majority being Saudi natives (n=981, 98.1%). Participants aged between 16 and 25 years constituted the largest age group (n=380, 38%), and more than half of the participants were female (n=557, 55.7%). Furthermore, the majority of participants held a university degree (n=547, 54.7%) in a non-medical major (n=733, 73.3%). The married category slightly exceeded the single category, with 48.4% (n=484) and 46.4% (n=464), respectively. In terms of geographical distribution, just under half of the study group resided in the Western regions of Saudi Arabia (n=480, 48%). Additionally, a significant portion of participants reported a high monthly income exceeding 10,000 Saudi Riyals, accounting for 49.7% (n=497) of the sample (Table 1).

**Table 1:** Descriptive statistics of sociodemographic variables (n=1000).

Variable		n (%)
Age (years)	16-25	380 (38.0)
	26-45	350 (35.0)
	46-65	270 (27.0)
Gender	Female	557 (55.7)
	Male	443 (44.3)
Marital status	Single	464 (46.4)
	Married	484 (48.4)
	Divorced	46 (4.6)
	Widowed	6 (0.6)
Education level	Illiterate	3 (0.3)
	Primary school	16 (1.6)
	Middle School	51 (5.1)
	High school	307 (30.7)
	Bachelor's degree	547 (54.7)
	Other	76 (7.6)
Field of work/study	Medical	267 (26.7)
	Non-medical	733 (73.3)
Family income (Saudi Riyal)	<5000	116 (11.6)
	5000-10000	387 (38.7)
	>10000	497 (49.7)
Nationality	Saudi	981 (98.1)
	Non-Saudi	19 (1.9)
Place of residence	Northern (Northern region, Jouf)	46 (4.6)
	Southern (Jazan, Najran, Asir)	66 (6.6)
	Center (Riyadh, Qassim, Hail)	218 (21.8)
	Eastern	150 (15.0)
	Western (Tabuk, Makkah, Madina, Al-Baha)	480 (48.0)
	Other	40 (4.0)

In the knowledge section, three questions were posed. The first question inquired whether the participants had prior knowledge of antimicrobial resistance, and the majority, specifically more than three-quarters (n=765, 76.5%), responded “Yes,” indicating their preexisting awareness of this medical concern.

The second question aimed to determine if participants were knowledgeable about the causes of antimicrobial resistance. Only a small fraction of participants (n=242, 24.2%) provided the correct response, acknowledging that both "taking antibiotics when there is no medical need for it" and "not completing the prescribed antibiotic course" contribute to antimicrobial resistance.

The third question sought to assess the participants' understanding of the true meaning of antimicrobial resistance. A significant proportion (n=694, 69.4%) correctly answered "Yes" to this question, indicating their understanding of the concept (Table 2).

**Table 2:** Responses to knowledge questions (n=1000). ^*^The accurate answer indicates a good level of knowledge about antimicrobial resistance.

Variables		n (%)
Question 1	Yes^*^	765 (76.5)
	No	235 (23.5)
Question 2	Taking antibiotics when there is no need for it	231 (23.1)
	Not finishing the prescribed antibiotic course	276 (27.6)
	Both^*^	242 (24.2)
	Don’t know	251 (25.1)
Question 3	Yes^*^	694 (69.4)
	No	42 (4.2)
	Don’t know	264 (26.4)

We also posed three questions to assess participants' attitudes toward the use of antibiotics in their daily lives. The first question inquired whether they used antibiotics as a preventive measure for diseases. Most of them (n=524, 52.4%) responded that they sometimes used antibiotics for this purpose, while only 29.2% (n=292) never used them. When participants were asked if they stored antibiotics at home and used them when needed, a significant portion (n=508, 50.8%) responded with "never."

The final question aimed to gauge the participants' antibiotic usage based on medical prescriptions. The results revealed that 33.3% (n=333) often and 47.3% (n=473) always followed medical prescriptions when using antibiotics. Notably, the questions’ answers, especially for the last question, with the highest mean of 3.25 (±0.83), suggested a strong awareness among participants regarding the proper use of antibiotics (Table 3).

**Table 3:** Responses to attitude questions (n=1000). To assess attitudes, Likert scales with the following four response options were employed: "never" (coded as 1), "sometimes" (coded as 2), "often" (coded as 3), and "always" (coded as 4). The collected data were analyzed by calculating the mean (±SD) response for each attitude question, utilizing the assigned numerical values.

Variables	Mean (±SD)	Never, n (%)	Sometimes, n (%)	Often, n (%)	Always, n (%)
Question 1	1.94 (±0.78)	292 (29.2)	524 (52.4)	134 (13.4)	50 (5.0)
Question 2	1.74 (±0.90)	508 (50.8)	311 (31.1)	116 (11.6)	65 (6.5)
Question 3	3.25 (±0.83)	30 (3.0)	164 (16.4)	333 (33.3)	473 (47.3)

Regarding the practices participants employ when it comes to the usage of antibiotics, we inquired about four key aspects. The first question pertained to the frequency of antibiotic usage, and slightly more than half (n=505, 50.5%) indicated that they rarely use antibiotics. A smaller percentage, 15.5% (n=155) mentioned using them once per year, and 20.4% (n=204) reported using them once every five to eight months. The second question investigated the source of antibiotic prescriptions. Notably, over two-thirds (n=688, 68.8%) reported that they received their antibiotic prescriptions from a doctor. However, it's worth highlighting that 23.4% (n=234) of participants reported obtaining antibiotic prescriptions from multiple sources besides doctors, including pharmacists, family members, or even engaging in self-prescription (Table 4).

**Table 4:** Responses to practice questions (n=1000). Statistical significance was defined as having a p-value of 0.05 or lower, and a 95% confidence interval was computed for the analyses.

Variables		n (%)
Question 1	More than once in a month	42 (4.2)
	Once every 2-4 months	94 (9.4)
	Once every 5-8 months	204 (20.4)
	Once in a year	155 (15.5)
	Rarely	505 (50.5)
Question 2	The doctor	688 (68.8)
	The pharmacist	45 (4.5)
	Self-prescription	24 (2.4)
	Family members or relatives	9 (0.9)
	Multiple sources	234 (23.4)
Question 4	After completing the course	689 (68.9)
	After feeling better	271 (27.1)
	Don’t know	40 (4.0)

**Figure 1:**
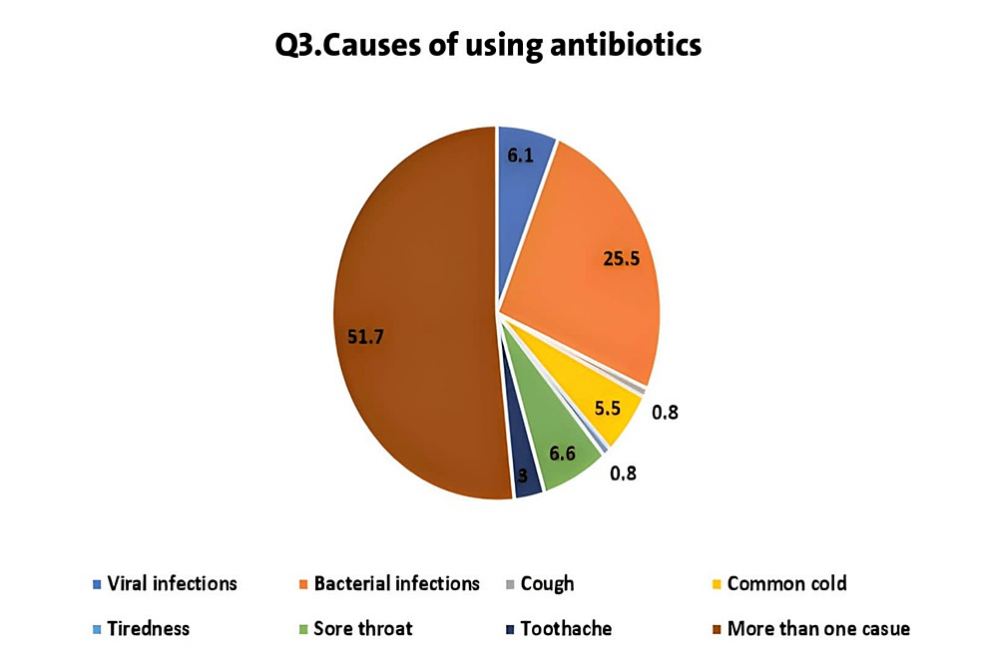
Indications for antibiotic usage reported by participants (n=1000).

The third question focused on the indications for which they use antibiotics. Surprisingly, participants reported a wide range of indications, including infections, cough, common cold, toothache, sore throat, and even tiredness. More than half of them (n=517, 51.7%) mentioned using antibiotics for more than one of the mentioned options. Only a small portion (n=255, 25.5%) stated that they exclusively used antibiotics for bacterial infections. It is notable; however, that 6.1% (n=61) still believed in using antibiotics to treat viral infections (Figure 1). This is a concerning finding, as the misuse and overuse of antibiotics are major contributors to antimicrobial resistance.

In the fourth question related to participants' practices concerning antimicrobial resistance, we inquired about when they typically stop taking antibiotics. The majority (n=689, 68.9%) indicated that they cease taking antibiotics once the treatment course is completed, which is a practice in line with preventing the development of antimicrobial resistance. However, it is worth noting that 27.1% (n=271) admitted to stopping antibiotics once they start feeling better, which may not align with best practices and can potentially contribute to the development of antimicrobial resistance (Table 4).

## Discussion

The issue of antibiotic resistance is a worldwide challenge, which is further exacerbated by the growing trend of inappropriate use of antibiotics in both medical environments and the general population. The Kingdom of Saudi Arabia has witnessed a rising incidence of extended-spectrum *Klebsiella pneumoniae *and *Escherichia coli *[15]. Saudi Arabia has a historical significance due to its role as the place of the annual Hajj pilgrimage, which has attracted a growing number of pilgrims in recent years. Additionally, being situated in the Middle East, it serves as an epicenter for antibiotic resistance. The use of antibiotics is influenced by the relationship of knowledge, expectations, and interactions between healthcare providers and patients, alongside economic, health systems, and environmental determinants [16]. Therefore, it is important to evaluate the general population's level of knowledge, attitudes, and practices (KAP) on the use of antibiotics. The present study revealed average knowledge (56.7%), adequately positive attitudes (61.3%), and inadequate practices (46.5%) towards antibiotic resistance. These findings are quite contrary to the results obtained in Kuwait and Malaysia [17,18]. Moreover, our findings are slightly higher than those found in a Jordan study, which reported a poor knowledge percentage (473%) [19].

Our study revealed that a significant proportion of participants (76.5%) had prior knowledge of antibiotic resistance. This level of awareness is encouraging, as it suggests that a considerable portion of the population is informed about the potential consequences of misuse and overuse of antibiotics. Only 27.6% of participants correctly identified that not completing the prescribed antibiotic course contributes to antibiotic resistance. This is in contrast to a prior survey that was conducted in 2021 throughout all five areas of Saudi Arabia, which reveals that 74.9% of participants understood that failing to complete an antibiotic's recommended course of treatment might result in antibiotic resistance [6]. Our study found a greater degree of knowledge compared to the findings published by El Zowalaty et al. in 2016. In their study, the reported levels of antibiotic use awareness were 48% [20].

The current survey demonstrated a satisfactory level of attitudes regarding the use of antibiotics, as shown by an overall attitude score of 61.3%. Significant proportions acknowledged that they used antibiotics as a preventive measure for diseases; sometimes 52.4%, often 13.4%, and always 5%, which suggests a prevalent misconception about the role of antibiotics in viral illnesses or non-bacterial conditions. However, only 29.2% believed that they never used antibiotics as a preventive method. On the positive side, a large portion of participants (50.8%) reported never storing antibiotics at home for self-use. This is a positive practice that aligns with wise antibiotic use. This finding is inconsistent with the results of previous studies conducted in Lithuania [21], Malaysia [18], and Saudi Arabia [22], where only 28.5%, 17%, and 25% of participants, respectively, reported storing antibiotics at home for future use. Moreover, according to research done by AlRukban et al. in 2020, it was found that almost two-thirds of the participants had antibiotics stored inside their households [23]. In terms of following medical prescriptions, the results are promising. A significant majority (80.6%) either often or always adhered to medical prescriptions when using antibiotics. These findings go in line with those conducted by Al-Shibani et al. in 2017, who reported that 82.2% of the participants took antibiotics on time [24]. This responsible behavior is crucial in preventing the development of antibiotic resistance. It is vital to promote and reinforce such practices to ensure that they remain consistent over time.

Regarding the respondents’ practices of antibiotic usage, it is reassuring to note that over half of the participants (50.5%) reported rarely using antibiotics. This suggests that a substantial portion of the population is not engaging in unnecessary antibiotic consumption. The finding that over two-thirds (68.8%) of participants received antibiotic prescriptions from doctors is encouraging. This underscores the importance of healthcare professionals' role in antibiotic stewardship. However, the fact that a significant minority obtained antibiotics from sources other than doctors is concerning. A study conducted among the general population residing in coastal south Karnataka, India, revealed that a significant proportion of participants (67%) had a favorable practice towards purchasing antibiotics on prescription only from a physician [25]. According to the survey conducted in Kuwait, around 64.7% of the participants expressed confidence in physicians to obtain antibiotics on a valid prescription [17]. A Saudi Arabian study reported that a significant proportion of respondents (72.6%) expressed agreement with the notion that antibiotics should not be obtained without a prescription [22]. According to the research conducted in Indonesia, around 40% of the participants reported purchasing antibiotics from pharmacies but without obtaining prescriptions [26]. Conversely, a majority (77.9%) disagreed with the idea of seeking a different physician solely due to their refusal to prescribe antibiotics. Furthermore, a substantial percentage (73.9%) disagreed with the practice of obtaining antibiotics from a drugstore without a valid prescription [22].

Two recent research studies conducted in Riyadh revealed that antibiotics are available for purchase without the need for a medical prescription from a physician [27,28]. Hence, the authors propose that this may be the underlying cause for the use of self-prescribed antibiotics within the examined group. Therefore, it is essential to conduct an investigation on the comparative significance of over-the-counter sales and the attitudes of pharmacists regarding antibiotics.

In our investigation, it was observed that the participants had difficulties in completing the course of prescribed antibiotics. It is worth noting that 27.1% of participants admitted to stopping antibiotics once they started feeling better, rather than completing the prescribed course. This percentage is a lot less than that of the Saudi Arabian study in which 66.8% of individuals successfully stopped the use of antibiotics after completing the course [22]. In another Indonesian study, 37% of participants did not stop the use of antibiotics until the full course was completed [26]. Regarding the purchase of antibiotics on prescription, antibiotics are reportedly being sold illegally by a large number of pharmacies in Saudi Arabia. In 2001, the research team for a study conducted in the eastern part of Saudi Arabia went to several different community pharmacies while reporting symptoms of urinary tract infections (UTIs). Eighty percent of the drugs handed out were antibiotics, and just one pharmacy out of 88 refused to give them out [29]. Another research in the Riyadh area used the same methodology for pharmacies in 2011. They discovered that 244 pharmacies (77%) out of 327 sampled did not follow the law while dispensing antibiotics [28]. Misconceptions regarding antibiotics and antimicrobial resistance caused by a lack of information contribute to the inappropriate use and overprescribing of antibiotics. If we don't restrain these harmful habits, it won't be long until untreatable infections begin to appear both locally and universally.

Limitations

Careful measures were made to get findings that are both accurate and exact, while also ensuring their accuracy. Nevertheless, this study has several limitations. The use of an online questionnaire for data collection is accompanied by inherent limitations. Furthermore, it should be noted that the sample size used in our research is quite small in relation to the precise number required for generalization. Hence, the findings of this study are limited only to the individuals involved, necessitating more research with a more extensive sample size. In addition, we acknowledge the fact that the cross-sectional design cannot establish causality but simply relationships. The present research also may have limited generalizability due to the use of convenience sampling. There are; however, inevitable drawbacks to all prior research like this due to a lack of casualties and restricted generalizability.

## Conclusions

This study provides insights into the KAP of antibiotic resistance among the general population in Saudi Arabia. While there is a relatively high level of awareness, there is room for improvement in understanding the causes of antibiotic resistance. Attitudes indicate a reasonable level of compliance with proper antibiotic use, with room for further education regarding preventive use. The practice of completing antibiotic courses aligns with best practices, though a significant portion still stops when symptoms improve. Our results highlight the knowledge gap regarding antibiotics and their appropriate usage. Therefore, these findings can inform targeted public health interventions to combat antibiotic resistance in Saudi Arabia. However, to make sure that society is moving in the right pathway, more research on this subject is necessary. The questionnaire in our study was made based on previous relevant studies conducted in Saudi Arabia, the authors of those studies were contacted.

## Appendices

**Table 5:** The sociodemographic data, level of knowledge, attitude, and practice were involved in the questionnaire.

Questionnaire	
1. Age	16-25 years
	26-45 years
	46-65 years
2. Gender	Male
	Female
3. Social status	Single
	Married
	Divorced
	Widowed
4. Educational level	Illiterate
	Primary school
	Intermediate school
	High school
	Bachelor’s degree
	Others
5. Family income	Below 5000
	Between 5000 and 10000
	More than 10000
6. Field of study or work	Medical field
	Non-medical field
7. Residence	Riyadh
	Mecca
	Medina
	Qassim
	Eastern region
	Asir
	Tabuk
	Hail
	Northern borders
	Jazan
	Najran
	Al-Baha
	Al Jouf
	Others
8. Nationality	Saudi
	Non-Saudi
9. Have you heard about antibiotic resistance?	Yes
	No
10. Cause of antimicrobial resistance	Taking antibiotics when there is no need for it
	Not finishing the prescribed antibiotic course
	I don’t know
11. Antibiotic resistance means the ability of bacteria to develop mechanisms that make them resistant to the effect of antibiotics	Yes
	No
	I don't know
12. Do you use antibiotics as a preventive method for diseases?	Never
	Sometimes
	Often
	Always
13. Do you store antibiotics at home and use them when it is needed?	Never
	Sometimes
	Often
	Always
14. Do you take antibiotics on time as the doctor’s prescription?	Never
	Sometimes
	Often
	Always
15. How many times do you use antibiotics?	More than once in a month
	Once every 2-4 months
	Once every 5-8 months
	Once in a year
	Rarely
16. Source of antibiotic prescription	The doctor
	The pharmacist
	Self-prescription
	Family members or relatives
17. Cause of using antibiotics	Viral infections
	Bacterial infections
	Cough
	Common cold
	Tiredness
	Sore throat
	Toothache
18. When do you stop taking antibiotics?	After completing the course
	After feeling better
	I don’t know

## References

[1] Adedeji WA (2016). The treasure called antibiotics. Ann Ib Postgrad Med.

[2] Davies J (2006). Are antibiotics naturally antibiotics?. J Ind Microbiol Biotechnol.

[3] Ribeiro da Cunha B, Fonseca LP, Calado CR (2019). Antibiotic discovery: where have we come from, where do we go?. Antibiotics (Basel).

[4] Prigitano A, Romanò L, Auxilia F, Castaldi S, Tortorano AM (2018). Antibiotic resistance: Italian awareness survey 2016. J Infect Public Health.

[5] Belkina T, Al Warafi A, Hussein Eltom E, Tadjieva N, Kubena A, Vlcek J (2014). Antibiotic use and knowledge in the community of Yemen, Saudi Arabia, and Uzbekistan. J Infect Dev Ctries.

[6] Alnasser AH, Al-Tawfiq JA, Ahmed HA (2021). Public knowledge, attitude and practice towards antibiotics use and antimicrobial resistance in Saudi Arabia: a web-based cross-sectional survey. J Public Health Res.

[7] Miyano S, Htoon TT, Nozaki I, Pe EH, Tin HH (2022). Public knowledge, practices, and awareness of antibiotics and antibiotic resistance in Myanmar: the first national mobile phone panel survey. PLoS One.

[8] Alhomoud F, Aljamea Z, Basalelah L (2018). "Antibiotics kill things very quickly" - consumers' perspectives on non-prescribed antibiotic use in Saudi Arabia. BMC Public Health.

[9] Nogueira-Uzal N, Zapata-Cachafeiro M, Vázquez-Cancela O, López-Durán A, Herdeiro MT, Figueiras A (2020). Does the problem begin at the beginning? Medical students' knowledge and beliefs regarding antibiotics and resistance: a systematic review. Antimicrob Resist Infect Control.

[10] Benmerzouga I, Al-Zammay SA, Al-Shammari MM, Alsaif SA, Alhaidan TM, Aljofan M (2019). Practices of patients consuming antibiotics and knowledge about antibiotic resistance in hail region - Saudi Arabia. Future Science OA.

[11] Sham KS, Lwin OM, Ali AA, Kishor KS (2019). Knowledge, attitude, and awareness of antibiotic resistance among medical students. Arch Med Health Sci.

[12] André M, Vernby A, Berg J, Lundborg CS (2010). A survey of public knowledge and awareness related to antibiotic use and resistance in Sweden. J Antimicrob Chemother.

[13] Kurdi S, Faran A, Eareeni E, Alhalal N, Joseph R, Wali H, Alshayban D (2020). Assessment of knowledge and attitude toward the new antibiotic dispensing law and its effect on antibiotic use in Saudi Arabia. Saudi Pharm J.

[14] Alqarni SA, Abdulbari M (2019). Knowledge and attitude towards antibiotic use within consumers in Alkharj, Saudi Arabia. Saudi Pharm J.

[15] Al-Tawfiq JA, Rabaan AA, Saunar JV, Bazzi AM (2020). Antimicrobial resistance of gram-negative bacteria: a six-year longitudinal study in a hospital in Saudi Arabia. J Infect Public Health.

[16] Al-Tawfiq JA, Stephens G, Memish ZA (2010). Inappropriate antimicrobial use and potential solutions: a Middle Eastern perspective. Expert Rev Anti Infect Ther.

[17] Awad AI, Aboud EA (2015). Knowledge, attitude and practice towards antibiotic use among the public in Kuwait. PLoS One.

[18] Lim KK, Teh CC (2012). A cross sectional study of public knowledge and attitude towards antibiotics in Putrajaya, Malaysia. South Med Rev.

[19] Shehadeh M, Suaifan G, Darwish RM, Wazaify M, Zaru L, Alja'fari S (2012). Knowledge, attitudes and behavior regarding antibiotics use and misuse among adults in the community of Jordan. A pilot study. Saudi Pharm J.

[20] El Zowalaty ME, Belkina T, Bahashwan SA (2016). Knowledge, awareness, and attitudes toward antibiotic use and antimicrobial resistance among Saudi population. Int J Clin Pharm.

[21] Pavydė E, Veikutis V, Mačiulienė A, Mačiulis V, Petrikonis K, Stankevičius E (2015). Public knowledge, beliefs and behavior on antibiotic use and self-medication in Lithuania. Int J Environ Res Public Health.

[22] Shatla M, Althobaiti FS, Almqaiti A (2022). Public knowledge, attitudes, and practices towards antibiotic use and antimicrobial resistance in the Western Region of Saudi Arabia. Cureus.

[23] AlRukban M, AlRuthia Y, Almasaoud M (2020). Community pharmacists' views of the enforced antibiotics dispensing law and its impact on oral antibiotics sales in Saudi Arabia. Risk Manag Healthc Policy.

[24] Al-Shibani N, Hamed A, Labban N, Al-Kattan R, Al-Otaibi H, Alfadda S (2017). Knowledge, attitude and practice of antibiotic use and misuse among adults in Riyadh, Saudi Arabia. Saudi Med J.

[25] Bhardwaj K, Shenoy MS, Baliga S, Unnikrishnan B, Baliga BS (2021). Knowledge, attitude, and practices related to antibiotic use and resistance among the general public of coastal south Karnataka, India - a cross-sectional survey. Clin Epidemiology Glob Health.

[26] Karuniawati H, Hassali MA, Suryawati S, Ismail WI, Taufik T, Hossain MS (2021). Assessment of knowledge, attitude, and practice of antibiotic use among the population of Boyolali, Indonesia: a cross-sectional study. Int J Environ Res Public Health.

[27] Al Rasheed A, Yagoub U, Alkhashan H, Abdelhay O, Alawwad A, Al Aboud A, Al Battal S (2016). Prevalence and predictors of self-medication with antibiotics in Al Wazarat Health Center, Riyadh City, KSA. Biomed Res Int.

[28] Bin Abdulhak AA, Altannir MA, Almansor MA (2011). Non prescribed sale of antibiotics in Riyadh, Saudi Arabia: a cross sectional study. BMC Public Health.

[29] Al-Ghamdi MS (2001). Empirical treatment of uncomplicated urinary tract infection by community pharmacist in the Eastern province of Saudi Arabia. Saudi Med J.

